# Facial nerve hemangioma in the middle ear

**DOI:** 10.31744/einstein_journal/2018RC4509

**Published:** 2018-11-29

**Authors:** Ludmilla Emilia Martins Costa, Rafael Freire de Castro, Fabiolla Maria Martins Costa, Mônica Alcântara de Oliveira Santos

**Affiliations:** 1 Hospital do Servidor Público Estadual “Francisco Morato de Oliveira, São Paulo, SP, Brazil.; 2 Centro Universitário do Maranhão, São Luís, MA, Brazil.

**Keywords:** Hemangioma, Facial nerve, Ear, middle, Hemangioma, Nervo facial, Orelha média

## Abstract

Facial nerve hemangioma is a rare and benign vascular tumor, and accounts for 0.7% of intratemporal tumors. We report the second case described in the literature of a facial nerve hemangioma in its tympanic segment. A 14-year-old male patient presented with a history of progressive right ear hearing loss with preserved facial mimicry. Pure tone audiometry showed a right ear moderate conductive hearing loss. Magnetic resonance imaging demonstrated an expansive lesion involving the tympanic segment of the right facial nerve, suggestive of hemangioma. Watchful waiting was chosen as management. In the first case of middle ear facial hemangioma described in the literature, facial palsy was the symptom that led the patient to seek medical care. In the present case, it can be inferred that the first symptom was conductive hearing loss ipsilateral to the lesion. Facial palsy may not be present and the clinical presentation may resemble otosclerosis, ossicular chain disruption, and third window abnormalities, among other differential diagnoses of conductive hearing loss. The second case of tympanic portion facial nerve hemangioma is reported, describing the specificity of conductive hearing loss as its only clinical manifestation.

## RESUMO

O hemangioma do nervo facial é um tumor vascular raro e benigno, que corresponde a 0,7% dos tumores intratemporais. Apresentamos o segundo caso descrito na literatura de hemangioma do nervo facial em sua porção timpânica. Paciente de 14 anos, sexo masculino, apresentando hipoacusia progressiva em orelha direita com mímica facial preservada. A audiometria evidenciou perda condutiva moderada à direita. A ressonância magnética demonstrou lesão expansiva, comprometendo o segmento timpânico do nervo facial direito, sugestiva de hemangioma do nervo facial. A conduta foi expectante. No primeiro caso descrito na literatura de hemangioma do facial em orelha média, o sintoma que levou o paciente ao atendimento médico foi paralisia facial. No presente caso, pode-se inferir que o primeiro sintoma foi a perda auditiva condutiva ipsilateral à lesão. A paralisia facial pode não estar presente, e o quadro clínico pode se assemelhar à otosclerose, disjunção de cadeia e síndrome da terceira janela, dentre outros diagnósticos diferenciais de perdas auditivas condutivas. O artigo relata o segundo caso de hemangioma do nervo facial em sua porção timpânica, mostrando a peculiaridade de perda auditiva condutiva como única manifestação clínica.

## INTRODUCTION

Hemangiomas are benign vascular tumors that may cause symptoms due to compression of adjacent structures. The tumors comprise a complex mixture of clonal endothelial cells with pericytes, dendritic cells and mast cells. Regulators of hemangioma growth and involution are not yet completely understood. During the hemangioma growth phase, two main pro-angiogenic factors are involved: fibroblast growth factor (FGF) and vascular endothelial growth factor (VEGF). Histological studies have shown that endothelial and interstitial cells actively divide at this stage.^(^ ^1^ ^)^

Facial nerve hemangioma is a rare and benign vascular tumor, account for 0.7% of intratemporal tumors. It originates from the arteriovenous plexus surrounding the facial nerve, and it is an important differential diagnosis of other temporal bone lesions that cause facial palsy, such as vestibular schwannoma and schwannoma of the facial nerve. Other differential diagnoses are glomus tumors, adenoma, meningioma, glioma, osteoma, dermoid cyst, cholesterol granuloma, among others.^(^ ^2^ ^)^

Facial nerve hemangiomas can occur in any segment of the facial nerve, and are more often located in the geniculate ganglion. We report the second case described in the literature of facial nerve hemangioma located in the tympanic portion of the facial nerve, and presenting conductive hearing loss as the only clinical manifestation. ^(^ ^3^ ^)^

## CASE REPORT

**Figure 1:**
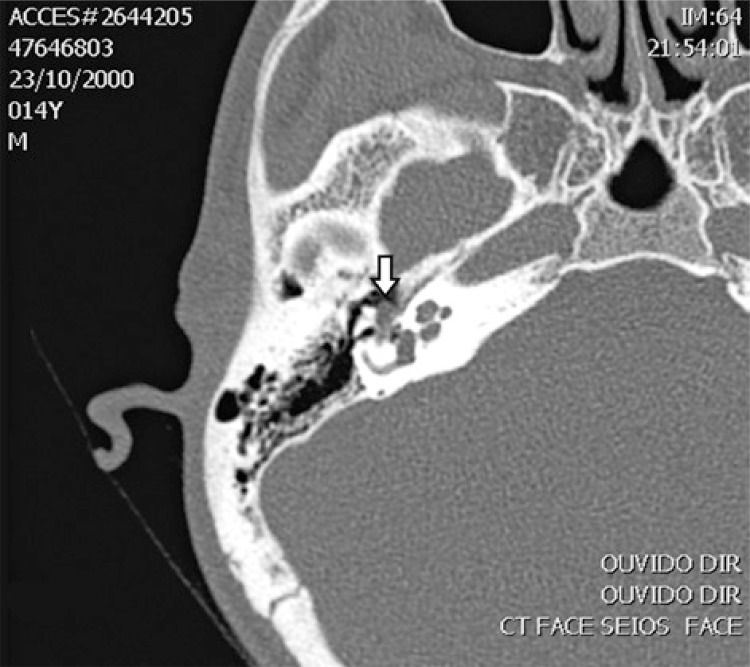
Computed tomography axial section view of temporal bones showing hemangioma (white arrow) of right facial nerve

**Figure 2:**
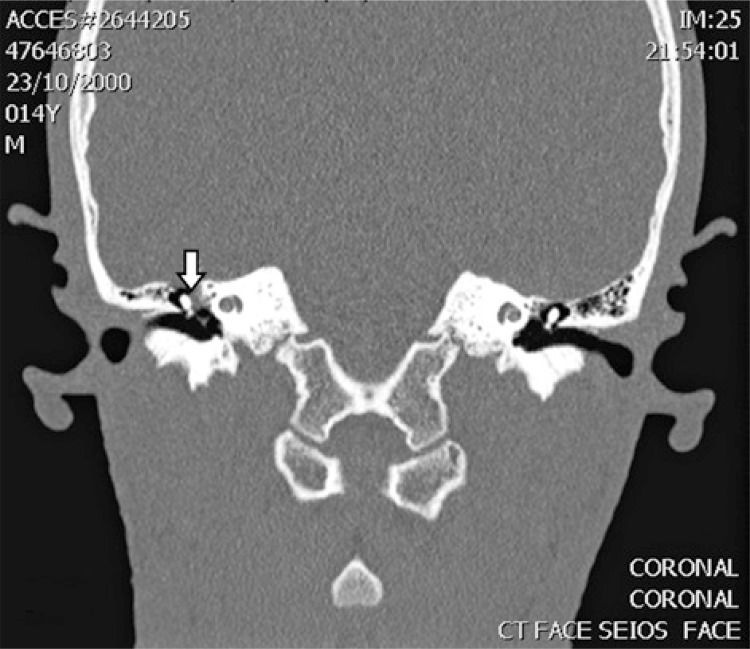
Computed tomography coronal section view of temporal bones showing hemangioma (white arrow) of right facial nerve

**Figure 3:**
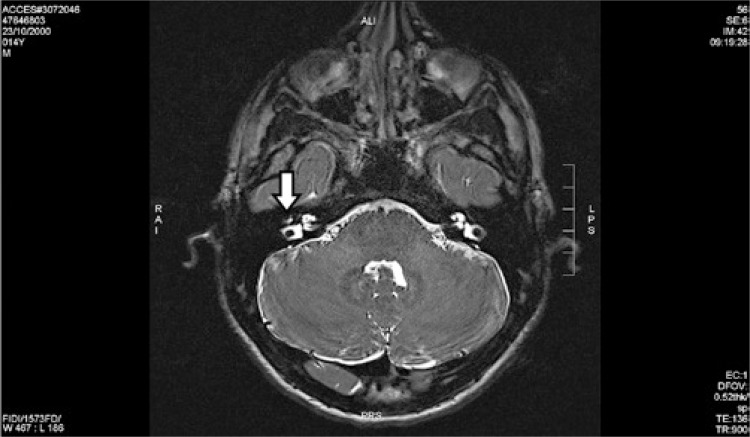
Axial T2-weighted cranial magnetic resonance imaging view showing hemangioma (white arrow) of the right facial nerve

**Figure 4:**
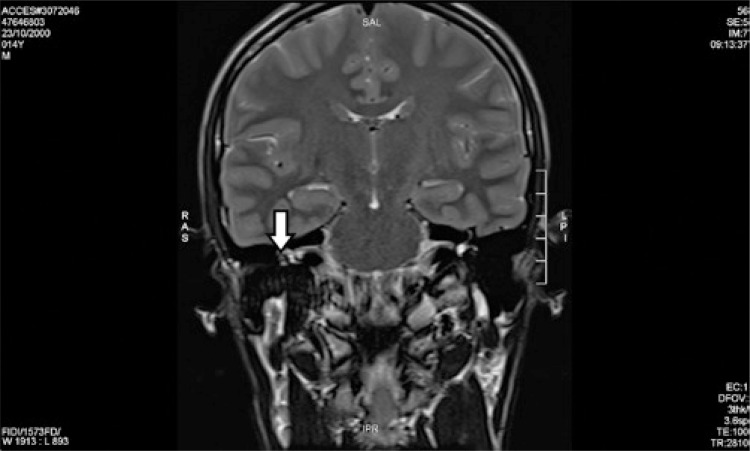
Coronal T2-weighted cranial magnetic resonance imaging view showing hemangioma (white arrow) of the right facial nerve

A 14-year-old male patient was presenting progressive hearing loss in the right ear for 2 years, learning disability and no previous medical conditions. Facial mimicry was preserved. Otoscopy showed intact and unaltered tympanic membranes. Audiometry revealed moderate right conductive loss, tympanometry with As curve to the right, curve A to the left and absence of contralateral acoustic reflex to the right. Computed tomography showed a round-shaped soft-tissue density lesion in the middle ear, close to facial nerve topography (Figures 1 and 2). Magnetic resonance imaging showed an expansive lesion with ill-defined contours, involving the tympanic segment of the right facial nerve, extending anteriorly to the level of the geniculate ganglion and, posteriorly, in its transition to the mastoid segment, with a slight attenuation by the paramagnetic contrast agent, suggestive of facial nerve hemangioma (Figures 3 and 4). Watchful waiting was the management chosen for the patient. After a 2-year follow-up, there was no worsening in hearing nor in facial mime impairment. Imaging studies did not reveal tumor growth.

## DISCUSSION

Facial nerve hemangiomas occur most commonly in the geniculate ganglion, while hemangiomas located in the tympanic portion of the facial nerve, as in the case reported, are more uncommon with only one case reported in the literature. The reason for hemangioma preference for the geniculate ganglion has not been well established, but studies by Balkany et al., with human temporal bones have suggested that the cause is likely to be the presence of high perineural capillary density in that portion of the facial nerve, in comparison with its other segments.^(^ ^4^ ^)^

The clinical presentation of patients with tumors of the temporal bone varies according to the type of tumor and its location. Some possible symptoms in patients with this neoplasm are progressive dysacusia, tinnitus, ear fullness, and facial palsy.

Hemangiomas in the geniculate ganglion region show progressive facial palsy (96%) by compression, associated with sensorineural hearing loss, more rarely (5%). Even in small tumors, these symptoms may already be present. Tumor expansion can involve the cochlea and result in pulsatile tinnitus. Facial palsy is less common when the lesion is located in the internal auditory canal (70%), and hearing loss is more prevalent in this location (90%).^(^ ^5^ ^)^ Few cases of facial nerve hemangioma in its mastoid or vertical portion have been reported and have shown variable facial symptoms associated with sensorineural or conductive hearing loss.^(^ ^6^ ^)^

The clinical presentation of patients with tympanic portion facial nerve hemangioma has not been well described, due to the rarity of the condition.^(^ ^7^ ^)^ However, according to the present case, it can be inferred that the first symptom, that often makes the patient seek the help of a specialist, is the conductive hearing loss ipsilateral to the lesion. Nonetheless, in the first case reported in the literature of middle ear facial nerve hemangioma, the symptom that led the patient to seek medical care was mild facial palsy and, during the investigation, mild conductive hearing loss was diagnosed.^(^ ^8^ ^)^

Since facial palsy may not be present, as in the present case, the clinical presentation may resemble otosclerosis, ossicular chain disruption, third window alterations, among other differential diagnoses of conductive hearing loss. Misdiagnosis, leading to untimely surgical management, could lead to serious consequences, such as hemorrhage and facial palsy.

The history, physical examination (especially otoscopy) and assessment of cranial nerve pairs help to diagnose the condition. The rare cases, such as the present, showing normal facial function are underdiagnosed. Audiometry is essential to assess the type of hearing loss. Tumor extension should be investigated by radiological examinations, such as computed tomography and magnetic resonance imaging.^(^ ^9^ ^)^ Facial nerve hemangiomas are generally hyperintense on T2-weighted images, and show intense gadolinium enhancement. Its T1-weighted intensity is variable.^(^ ^2^ ^)^

As facial nerve hemangioma in the tympanic segment is a very rare vascular tumor, there is no consensus as to its management. Propranolol, surgical excision and watchful waiting are possible therapeutic options for hemangiomas from other locations. More conservative management is the most appropriate choice for patients with preserved facial function.^(^ ^10^ ^)^

In other middle ear hemangiomas, not deriving from the facial nerve, surgical treatment is best indicated. The decision on which surgical technique to use depends on the size and location of the tumor, as well as on preoperative auditory function.^(^ ^8^ ^)^

Facial nerve hemangioma should be remembered as a differential diagnosis for ear tumors, as a cause of facial palsy and also conductive hearing losses, and not just for sensorineural hearing loss.

## CONCLUSION

This case report describes the second case of facial nerve hemangioma in the tympanic portion, showing the specificity of conductive hearing loss as the only clinical manifestation.
